# Total NT-proBNP, a novel biomarker related to recurrent atrial fibrillation

**DOI:** 10.1186/s12872-021-02358-y

**Published:** 2021-11-19

**Authors:** Lidia Staszewsky, Jennifer M. T. A. Meessen, Deborah Novelli, Ursula-Henrike Wienhues-Thelen, Marcello Disertori, Aldo P. Maggioni, Serge Masson, Gianni Tognoni, Maria Grazia Franzosi, Donata Lucci, Roberto Latini

**Affiliations:** 1 Department of Cardiovascular Medicine, Istituto Di Ricerche Farmacologiche Mario Negri IRCCS, Via Mario Negri 2, 20156 Milan, Italy; 2 Roche Diagnostics GmbH, Penzberg, Germany; 3 Healthcare Research and Innovation Program, IRCS-HTA, BK Foundation, Trento, Italy; 4 ANMCO Research Center, Florence, Italy; 5 Roche Diagnostics International Ltd, Rotkreuz, Switzerland; 6 Istituto Di Anestesia E Rianimazione, Università Degli Studi Di Milano, Ospedale Maggiore, Istituto Di Ricovero E Cura a Carattere Scientifico, Milan, Italy

**Keywords:** Atrial fibrillation, biomarkers, Brain natriuretic peptides, Recurrence, Cardiovascular hospitalization

## Abstract

**Background:**

Novel circulating biomarkers may help in understanding the underlying mechanisms of atrial fibrillation (AF), a challenge for AF management and prevention of cardiovascular (CV) events. Whether glycosylation affects the prognostic value of N-terminal pro-B type natriuretic peptide (NT-proBNP) in AF is still unknown.

**Objectives:**

To test how deglycosylated total NT-proBNP, NT-proBNP and a panel of biomarkers are associated with: (1) recurrent AF, (2) first hospitalization for CV reasons.

**Methods:**

A total of 382 patients of the GISSI-AF trial in sinus rhythm with a history of AF, echocardiographic variables, total NT-proBNP, NT-proBNP and nine additional biomarkers [Total N-terminal pro-B type natriuretic peptide (Total NT proBNP), N-terminal pro-B type natriuretic peptide (NTproBNP), Angiopoietin 2 (Ang2), Bone morphogenic protein-10 (BMP10), Dickkopf-related protein-3 (DKK3), Endothelial cell specific molecule-1 (ESM1), Fatty acid-binding protein 3 (FABP3), Fibroblast growth factor 23 (FGF23), Growth differentiation factor-15 (GDF15), Insulin-like growth factor-binding protein-7 (IGFBP7) and Myosin binding protein C3 (MYPBC3)]. were assayed at baseline, 6 and 12 months under blind conditions in a laboratory at Roche Diagnostics, Penzberg, Germany. The associations between circulating biomarkers and AF at the 6- and 12-month visits, and their predictive value, were assessed in multivariable models with logistic regression analysis and Cox proportional hazards regression analysis. Biomarkers associations were modelled for 1SD increase in their level.

**Results:**

Over a median follow-up of 365 days, 203/382 patients (53.1%) had at least one recurrence of AF and 16.3% were hospitalized for CV reasons. Total NT-proBNP, NT-proBNP, Ang2 and BMP10 showed the strongest associations with ongoing AF. Natriuretic peptides also predicted recurrent AF (total NT-proBNP: HR:1.19[1.04–1.36], p = 0.026; NT-proBNP: HR:1.19[1.06–1.35], p = 0.016; Ang2: HR:1.07[0.95–1.20], p = 0.283; BMP10: HR:1.09[0.96–1.25], p = 0.249) and CV hospitalization (total NT-proBNP: HR:1.57[1.29–1.90], p < 0.001 1.63], p = 0.097).

**Conclusions:**

The association of total NT-proBNP with the risk of AF first recurrence was similar to that of NT-proBNP, suggesting no influence of glycosylation. Analogous results were obtained for the risk of first hospitalization for CV reasons. Natriuretic peptides, Ang2 and BMP10 were associated with ongoing AF. Findings from the last two biomarkers point to a pathogenic role of cardiac extracellular matrix and cardiomyocyte growth in the myocardium of the right atrium and ventricle.

**Supplementary Information:**

The online version contains supplementary material available at 10.1186/s12872-021-02358-y.

## Background

Knowledge about atrial fibrillation (AF) has been steadily increasing over the last two decades together with the awareness that this arrhythmia is an important health problem [1]. Clinical and bio-humoral markers associated with AF increase our understanding of its mechanisms and may help in predicting the risk of recurrence of AF [2, 3]. Previous data from GISSI-AF trial and also from other authors showed that circulating biomarkers are associated to AF but are not strong predictors for AF recurrence in patients in sinus rhythm with a recent history of paroxysmal or persistent AF [4–6].

N-terminal pro-B type natriuretic peptide (NT-proBNP) has been regularly reported to be a strong—possibly the strongest—predictor of recurrent AF among several novel circulating biomarkers, as recently pointed out in the Framingham Heart Study [7]; however, its predictive power is still modest.

NT-proBNP and BNP are produced in equimolar amounts in cardiomyocytes in response to increased wall stretch, volume overload and ischemia (6–8); BNP but not NT-proBNP has physiological activity. There are nine known O-glycosylation sites on proBNP and NT-proBNP. On average, 7.4% of circulating NT-proBNP in HF patients is glycosylated in the central region of the molecule [11]. Commercial NT-proBNP ELISA contains antibodies directed to epitopes in the central region of NT-proBNP and detects non-glycosylated forms. Thus the assay underestimates the circulating concentrations of NT-proBNP when these sites are glycosylated [12, 13], and this may have different impacts on NT-proBNP’s performance as a biomarker in different pathologies [11].

The subgroup of patients in the bio-humoral substudy of GISSI-AF [14, 15] was deemed adequate to test independently two features of an exploratory panel of nine circulating biomarkers, deglycosylated total NT-proBNP and NT-proBNP in AF: (1) the association of a biomarker with AF, when a blood sample is taken at the 6- or 12-month visit while AF is present in the electrocardiogram (ECG), and (2) the predictive power of the same biomarker at baseline, when the patient is in sinus rhythm, for recurrence of AF or incident hospitalization for CV reasons. We focused on total NT-proBNP to assess whether glycosylation influences its prognostic accuracy.

## Methods

The GISSI-AF trial *(Clinical Trials.gov identifier: NCT00376272; EudraCT Number: 2004-003036-53)* was a double-blind randomized placebo-controlled multicenter trial in 114 cardiology divisions between November 2004–January 2007 that enrolled, 1442 patients in sinus rhythm with a history of AF (two or more episodes of symptomatic ECG-documented AF in the previous 6 months) or successful cardioversion, electrical or pharmacologic, between 14 days and 48 h before randomization). A routine clinical examination, including ECG and laboratory testing, was done at each study visit (baseline, weeks 2, 4, 8, 24 and 52). To increase the likelihood of detecting AF, all patients were given a trans-telephonic monitoring device (see Additional file 1: Appendix 1). Each AF episode during the trial was adjudicated blindly by a central reader and verified by an ad-hoc validation committee. The rationale, design, and results of the trial have already been published [14, 15]*.* Patients from 36 centers participated in a sub-study with serial bio-humoral tests at baseline, 6 and 12 months.

Ongoing AF is defined as the presence of AF rhythm in a 12- lead electrocardiogram recorded during the scheduled 6 or 12- month follow-up visit, during which a blood sample was drawn to assess circulating biomarkers. AF recurrence is defined as an episode of AF detected by telemonitoring or during the scheduled follow-up visits. In the first this case the patient was asked to come for an office visit to confirm the arrhythmia by a 12-lead ECG (see also Additional file 1: Appendix 1 Detection of recurrent AF during follow-up).

### Assays of circulating biomarkers and detection of AF recurrence

The following biomarkers were included in the analyses: total N-terminal pro-B type natriuretic peptide (total NT proBNP), N-terminal pro-B type natriuretic peptide (NT-proBNP), angiopoietin 2 (Ang2), bone morphogenic protein-10 (BMP10), Dickkopf-related protein-3 (DKK3), endothelial cell specific molecule-1 (ESM1), fatty acid-binding protein 3 (FABP3), fibroblast growth factor 23 (FGF23), growth differentiation factor-15 (GDF15), insulin-like growth factor-binding protein-7 (IGFBP7), and myosin binding protein C3 (MYPBC3). The characteristics of the biomarkers (e.g. an exploratory analysis of nine circulating biomarkers, total NT-proBNP and NT-proBNP), are described in Additional file 1: Appendix 2 Assays of circulating biomarkers and detection of recurrent AF during follow-up, and detectability in Table 1*.*

**Table 1:** Biomarker concentrations at each study visit and lower limit of quantification and detection

Biomarker	Visit	n	Mean	Std Dev	Median	Q1	Q3	Min	Max	**LoQ**	**LoD**
Total NT proBNP * pg/mL	BL	376	1727	1712	1230	667	2011	75	12,700	8.3 pg/mL	6.52 pg/mL
	6 M	321	1494	1611	985	507	1959	34	12,626		
	12 M	323	1672	1825	1008	564	2112	62	11,992		
NT-proBNP (pg/mL)	BL	382	344.6	496.3	191.0	95.0	367.0	5.0	4347	50 pg/mL#	10 pg/mL##
	6 M	325	278.6	414.5	136.0	66.0	324.0	5.0	3906		
	12 M	331	328.0	555.9	149.0	63.0	350.0	5.0	6345		
Ang2 * ng/mL	BL	379	3.44	1.95	2.91	2.21	3.99	0.73	15.04	0.058 ng/mL	0.028 ng/mL
	6 M	324	2.85	1.34	2.49	2.01	3.19	1.01	10.14		
	12 M	329	2.88	1.31	2.56	2.02	3.28	1.16	10.09		
BMP10 * ng/mL	BL	375	2.09	0.52	2.02	1.75	2.33	1.14	5.04	0.009 ng/mL	0.003 ng/mL
	6 M	321	2.05	0.47	2.01	1.75	2.28	1.16	4.76		
	12 M	327	2.09	0.50	2.00	1.74	2.34	1.15	4.54		
DKK3 * ng/mL	BL	377	59.87	15.37	57.5	49.3	68.7	27.1	142.6	0.025 ng/mL	0.003 ng/mL
	6 M	321	57.83	14.42	55.8	48.2	64.0	28.7	118.7		
	12 M	324	59.51	16.47	57.5	48.6	66.2	26.8	136.9		
ESM1 * ng/mL	BL	377	2.23	1.01	2.02	1.65	2.55	0.87	9.17	< 0.003 ng/mL	0.001 ng/mL
	6 M	321	2.04	0.65	1.94	1.62	2.33	0.97	5.65		
	12 M	324	2.13	0.70	1.99	1.64	2.47	0.85	5.68		
FABP3 * ng/mL	BL	375	30.98	10.96	29.2	24.2	35.3	12.1	105.3	1.0 ng/mL	n.a
	6 M	321	33.17	11.17	31.2	25.7	38.8	11.6	86.7		
	12 M	327	34.80	12.13	33.1	26.2	40.1	15.0	91.4		
FGF23 * pg/mL	BL	374	0.14	0.10	0.11	0.09	0.15	0.01	0.87	0.004 ng/mL	n.a
	6 M	321	0.14	0.09	0.11	0.09	0.15	0.01	0.92		
	12 M	322	0.14	0.10	0.12	0.09	0.15	0.03	0.94		
GDF15 pg/mL	BL	375	1272	793	1053	745	1523	235	7059	≤ 400 pg/mL	≤ 400 pg/mL
	6 M	321	1344	1136	1080	794	1518	317	12,471		
	12 M	327	1355	965	1140	808	1555	369	9783		
IGFBP7 * ng/mL	BL	379	178	44	172	152	194	97	685	0.4 ng/mL	0.01 ng/mL
	6 M	324	179	48	170	154	191	80	705		
	12 M	329	184	49	174	157	199	91	701		
MYBPC3 * ng/mL	BL	373	5.63	6.33	3.69	2.29	6.35	0.34	48.48	2.1 pg/mL	0.458 pg/mL
	6 M	321	4.86	6.31	3.18	1.98	5.42	0.29	66.73		
	12 M	327	4.98	5.91	3.10	1.91	5.95	0	47.05		

NT-proBNP—N-terminal pro-B type natriuretic peptide; Ang2—angiopoietin 2; BMP10—bone morphogenic protein-10; DKK3—Dickkopf-related protein-3; ESM1—endothelial cell specific molecule 1; FABP3—fatty acid-binding protein 3; FGF23—fibroblast growth factor 23; GDF15—growth differentiation factor-15; IGFBP7—insulin-like growth factor-binding protein-7; MyBPC3—myosin binding protein C3. Reference value: NT-proBNP, 125 pg/mL; GDF15, > 1500 ng/mL (arbitrary value suitable for GISSI-AF population). * Research Use Only (RUO) assays without full in vitro diagnostic (IVD) documentation, reference value not available. LoQ, lower limit of quantification; LoD, lower limit of detection. #, LoQ – 20% CV at ≤ 50 pg/mL C; ##, 10 pg/mL (specification) on cobas e 411, cobas e 601, cobas e 602;

Per protocol, for handling and processing the samples were left in the local lab for up to 1 h before freezing at − 70 °C. Samples stored locally were transferred to the core lab every year.

Biomarkers were assayed under blind conditions in a laboratory at Roche Diagnostics, Penzberg, Germany. Total NT-proBNP (Roche Diagnostics GmbH, Mannheim) was measured on a Cobas Elecsys Immunoanalyzer with a prototype sandwich immunoassay for use in exploratory research. This detects any NT-proBNP that is not O-glycosylated (position S44). The lower limit of detection is 6.5 pg/mL; within-run and between-run precisions are ≤ 1.2% and ≤ 2.5%.

### Statistical methods

Primary objective of this study is to evaluate whether deglycosylated total NT-proBNP, NT-proBNP and other nine circulating biomarkers are associated to AF, both ongoing or recurrent; the secondary endpoint is the relationship with first hospitalization for cardiovascular reasons. Continuous variables are expressed as mean ± SD if normally distributed or median and interquartile range [IQR] if not normally distributed; categorical variables were reported as absolute numbers and percentages. Differences between groups of patients with and without ongoing or recurrent AF were assessed with one-way ANOVA, Kruskal–Wallis test or χ^2^ test, as appropriate. The correlations between circulating biomarkers were analyzed with Spearman's rank correlation coefficient.

The association of each biomarker with ongoing AF at the follow-up visit was assessed on the basis of the area under the curve (AUC) of ROC analysis, followed by logistic regression analysis adjusted for variables significantly associated with AF in univariate analysis: heart failure, LVEF < 40% or both, history of hypertension, AF episode with LA dilatation, and oral anticoagulant use. Biomarkers which had an AUC < 70% and were not independently associated with ongoing atrial fibrillation were then excluded from further analyses except BMP10. This biomarker was carried included in the analyses after an authoritative study on its mechanistic involvement in the pathophysiology of AF and its specific production by atrial tissue [16]

Kaplan–Meier curves and log-rank tests were used to assess differences in the AF recurrence-free survival according to biomarker baseline values above or below the median. Biomarkers were modelled as continuous variables (expressed as 1 SD increment) as linearity was tested by restricted cubic splines. Cox proportional univariable and adjusted hazard models were constructed to assess the prediction of AF recurrence. Cardiovascular hospitalization was predicted similarly. Cox analyses were adjusted for covariates selected on the basis of univariate analysis; for AF recurrence, sex and two or more episodes of AF in the six months before inclusion in GISSI-AF. For cardiovascular hospitalization, systolic blood pressure, history of hypertension, peripheral artery disease and smoking. The c-index derived from the multivariable models was used to assess the improvement in the prognostic model including either Total NT-proBNP or NT-proBNP in the adjusted model. Comparisons between the areas under the ROC curves were performed with the use of U-statistics [17]. All probability values are two-tailed and p-values were corrected for multiple testing by means of the False Discovery Rate (FDR-correction). A p < 0.05 was considered significant. Data were analyzed using SPSS Version 25 (IBM SPSS, Armonk, NY) and SAS Version 9.4.

## Results

### Patients

The study comprised 382 patients, and 1038 plasma samples were analyzed (baseline, 382; six-month follow-up 325; 12-month follow-up 331). The baseline characteristics of this population were similar to those of the 1442 patients enrolled in the main GISSI-AF study [15]. In brief, age was 67.6 ± 9.1 years, 142 (37.2%) were women; 154 (40.8%) had had two or more episodes of AF in the previous 6 months and 336 (88.0%) had undergone cardioversion for AF in the previous two weeks. There was a history of hypertension in 324 (84.8%), history of stroke 15 (3.9%), and heart failure (HF) or LVEF < 40% in 42 (11.0%). The study treatment, valsartan, was given to 186 patients (48.7%), ACE-inhibitors to 206 (53.9%), beta-blockers to 114 (29.8%) and aldosterone blockers to 20 (5.2%) (Additional file 1: Table 1 Baseline clinical, eletrocardiographic and echocardiograpic characteristics of all patients and according to ongoing AF and AF recurrence).

During follow-up (median 365 days, range 5–373 days), 203 (53.1%) patients had at least one newly diagnosed episode of AF and 113 (29.6%) had more than one. The median number of episodes of recurrent AF per patient was 3 (range 2–27). During the one-year follow-up one patient died, and the incidence of hospitalization was 18.7% for any reason and 16.3% for CV reasons. As there were no significant differences between valsartan and placebo for any of the circulating biomarkers and echocardiographic variables, the whole cohort of 382 patients was analyzed, irrespective of the treatment.

### Plasma concentrations of circulating biomarkers at baseline and during follow-up

Concentrations of total NT-proBNP and the other biomarkers at baseline and at each visit are shown in Table 1; medians were within the reference range except for total NT-proBNP and NT-pro BNP, where the levels were above the normal range. Median concentrations of all biomarkers were either stable or decreased slightly over the 12-month follow-up. Linear correlations between different biomarkers showed r values ranging from 0.90 for total NT-proBNP—NT-proBNP, to 0.11 for Ang2—FABP2 (Additional file 1: Table 2 Correlation coefficients for biomarker concentrations at baseline).

### Association between circulating biomarkers and ongoing AF

At 6- and 12-months follow-up, respectively 34/325 (10.5%) and 45/331 (13.6%) patients had ongoing AF in the 12-lead ECG. Concentrations of total NT-proBNP, NT-proBNP, Ang2, BMP10, DKK3, FGF23 and MyBPC3 were significantly higher in those with AF at both visits. Natriuretic peptides and Ang2 showed by far the largest significant increases in the patients with AF at 6 and 12 months (P < 0.001) (Table 2).

**Table 2:** Concentrations of circulating biomarkers in patients with sinus rhythm or with atrial fibrillation recorded by ECG during scheduled visits at 6 and 12 months

Biomarker	Visit	Rhythm	n	Median	IQR	AUC	95%CI	P_fdr_
Total NT-proBNP (pg/mL)	6 months	Sinus	280	865	[484–1720]	0.777	(0.682–0.871)	6.1 × 10^–6^
		AF	34	2532	[1504–4692]			
	12 months	Sinus	268	878	[476–1809]	0.772	(0.705–0.838)	3.0 × 10^–7^
		AF	45	2241	[1381–4032]			
NT-proBNP (pg/mL)	6 months	Sinus	284	128.0	[55.3–238.5]	0.866	(0.785–0.947)	3.3 × 10^–10^
		AF	34	724.5	[435.8–1311.8]			
	12 months	Sinus	276	116.5	[54.0–242.5]	0.862	(0.806–0.918)	1.5 × 10^–12^
		AF	45	580.0	[306.0–1180.0]			
Ang2 (ng/mL)	6 months	Sinus	284	2.44	[1.95–3.03]	0.726	(0.636–8.16)	0.001
		AF	34	3.27	[2.38–4.95]			
	12 months	Sinus	274	2.39	[1.94–3.07]	0.816	(0.754–0.877)	8.8 × 10^–10^
		AF	45	3.75	[2.96–5.18]			
BMP10 (ng/mL)	6 months	Sinus	281	1.97	[1.72–2.23]	0.710	(0.611–0.810)	2.8 × 10^–4^
		AF	33	2.31	[2.04–2.67]			
	12 months	Sinus	273	1.97	[1.73–2.27]	0.634	(0.537–0.731)	0.007
		AF	44	2.18	[1.91–2.65]			
DKK3 (ng/mL)	6 months	Sinus	280	55.25	[47.27–63.06]	0.640	(0.541–0.738)	0.013
		AF	34	59.94	[53.64–78.79]			
	12 months	Sinus	269	46.45	[48.20–64.09]	0.648	(0.558–0.737)	0.002
		AF	45	62.66	[53.99–77.87]			
ESM1 (ng/mL)	6 months	Sinus	280	1.92	[1.61–2.31]	0.601	(0.490–713)	0.067
		AF	34	2.07	[1.71–2.88]			
	12 months	Sinus	269	1.96	[1.61–2.38]	0.623	(0.532–0.714)	0.013
		AF	45	2.27	[1.87–2.80]			
FABP3 (ng/mL)	6 months	Sinus	281	30.86	[25.62–38.45]	0.597	(0.495–0.699)	0.079
		AF	33	33.74	[26.86–44.43			
	12 months	Sinus	273	33.11	[26.50–40.47]	0.457	(0.359–0.554)	0.380
		AF	44	30.37	[24.61–39.34]			
FGF23 (ng/mL)	6 months	Sinus	281	0.11	[0.09–0.14]	0.716	(0.625–0.807)	0.001
		AF	33	0.15	[0.12–0.19]			
	12 months	Sinus	270	0.11	[0.09–0.15]	0.617	(0.525–0.708)	0.019
		AF	44	0.14	[0.10–0.18]			
GDF15 (pg/mL)	6 months	Sinus	281	1058	[791–1446]	0.602	(0.493–0.712)	0.067
		AF	33	1440	[883–1715]			
	12 months	Sinus	273	1140	[812–1554]	0.508	(0.416–0.599)	0.870
		AF	44	1143	[796–1598]			
IGFBP7 (ng/mL)	6 months	Sinus	284	167.94	[153.8–186.8]	0.665	(0.564–0.766)	0.004
		AF	34	187.07	[164.6–230.4]			
	12 months	Sinus	274	172.41	[156.0–196.7]	0.542	(0.450–0.634)	0.380
		AF	45	174.74	[159.8–210.4]			
MYBPC3 (pg/mL)	6 months	Sinus	281	3.09	[1.95–5.17]	0.656	(0.559–0.753)	0.006
		AF	33	4.62	[2.63–9.87]			
	12 months	Sinus	273	2.98	[1.79–5.28]	0.609	(0.524–0.695)	0.028
		AF	44	4.00	[2.48–6.68]			

Biomarker concentration at 6 and 12 months and results of ROC-analysis for discriminatory value to predict AF. P value from multiple testing by means of FDR-correction. NT-proBNP—N-terminal pro-B type natriuretic peptide; Ang2—angiopoietin 2; BMP10—bone morphogenic protein-10; DKK3—Dickkopf-related protein-3; ESM1—endothelial cell specific molecule 1; FABP3—fatty acid-binding protein C3; FGF23—fibroblast growth factor 23; GDF15—growth differentiation factor-15; IGFBP7—insulin like growth factor-binding protein-7; MyBPC3—myosin binding protein 3

ROC analyses were done to assess the strength of the relation between biomarkers and ongoing AF (i.e. biomarkers assayed while an AF rhythm was recorded in a 12-lead ECG during a clinical visit). The AUCs were highest at 6 and 12 months, (Table 2) for total NT-proBNP (AUC_6M_ 0.78 and AUC_12M_ 0.77), NT-proBNP (AUC_6M_ 0.87 and AUC_12M_ 0.86), Ang2 (AUC_6M_ 0.73 and AUC_12M_ 0.82); for BMP10 AUC_6M_ was 0.71 and AUC_12M,_ 0.63. Multivariable logistic regression models estimated the association between these four biomarkers (total NT-proBNP, NT-proBNP, Ang2 and BMP10) with ongoing AF. The association was significant for all four after adjustment for clinical variables that were significant in univariable analysis (Table 3).

**Table 3:** Biomarkers associated with ongoing atrial fibrillation

	Univariate model		Multivariable model	
Biomarker	OR (95%CI)	P_fdr_	OR (95%CI)	P_fdr_
Total NT-proBNP	1.70 (1.32–2.19)	**7.4 × 10**^**–5**^	1.58 (1.21–2.07)	**0.001**
NT-proBNP	2.19 (1.66–2.88)	**1.2 × 10**^**–7**^	2.02 (1.52–2.70)	**8.0 × 10**^**–6**^
Ang2	1.43 (1.13–1.82)	**0.004**	1.32 (1.02–1.71)	**0.034**
BMP10	1.60 (1.23–2.09)	**6.0 × 10**^**–4**^	1.56 (1.19–2.04)	**0.001**

The association between biomarker concentration and AF during clinical visits was assessed by means of logistic regression models. The biomarker concentration was the value while AF was ongoing (i.e. if a patient presented AF at the six-month visit, the corresponding biomarker value was included while if the AF was present at 12 months that value was included). If no AF arose during visits, the baseline value was inserted. Data shown as odds ratio (OR) for an increment of 1 SD of baseline

Multivariable model was adjusted for: heart failure, LVEF < 40% or both, history of hypertension and AF episode with LA dilatation. P-value was corrected for multiple testing by means of FDR-correction

NT-proBNP, N-terminal pro-B type natriuretic peptide; Ang2, angiopoietin 2; BMP10, bone morphogenic protein-10

### Circulating biomarkers as predictors of AF recurrence

Total NT-proBNP, NT-proBNP, Ang2 and BMP10 were then included in analyses for the prediction of AF recurrence. In one year of follow-up, 203 patients (53.1%) experienced at least one episode of AF recurrence, mostly identified in telemetric device recordings. Clinical characteristics and treatments of patients with at least one AF recurrence and with any recurrence were previously published [5] (see also Additional file 1: Table 1 Baseline clinical, eletrocardiographic and echocardiograpic characteristics of all patients and according to ongoing AF and AF recurrence). Males and patients with more than two episodes of AF in the six months prior to inclusion in the study were more likely to suffer at least one AF recurrence.

**Fig. 1:**
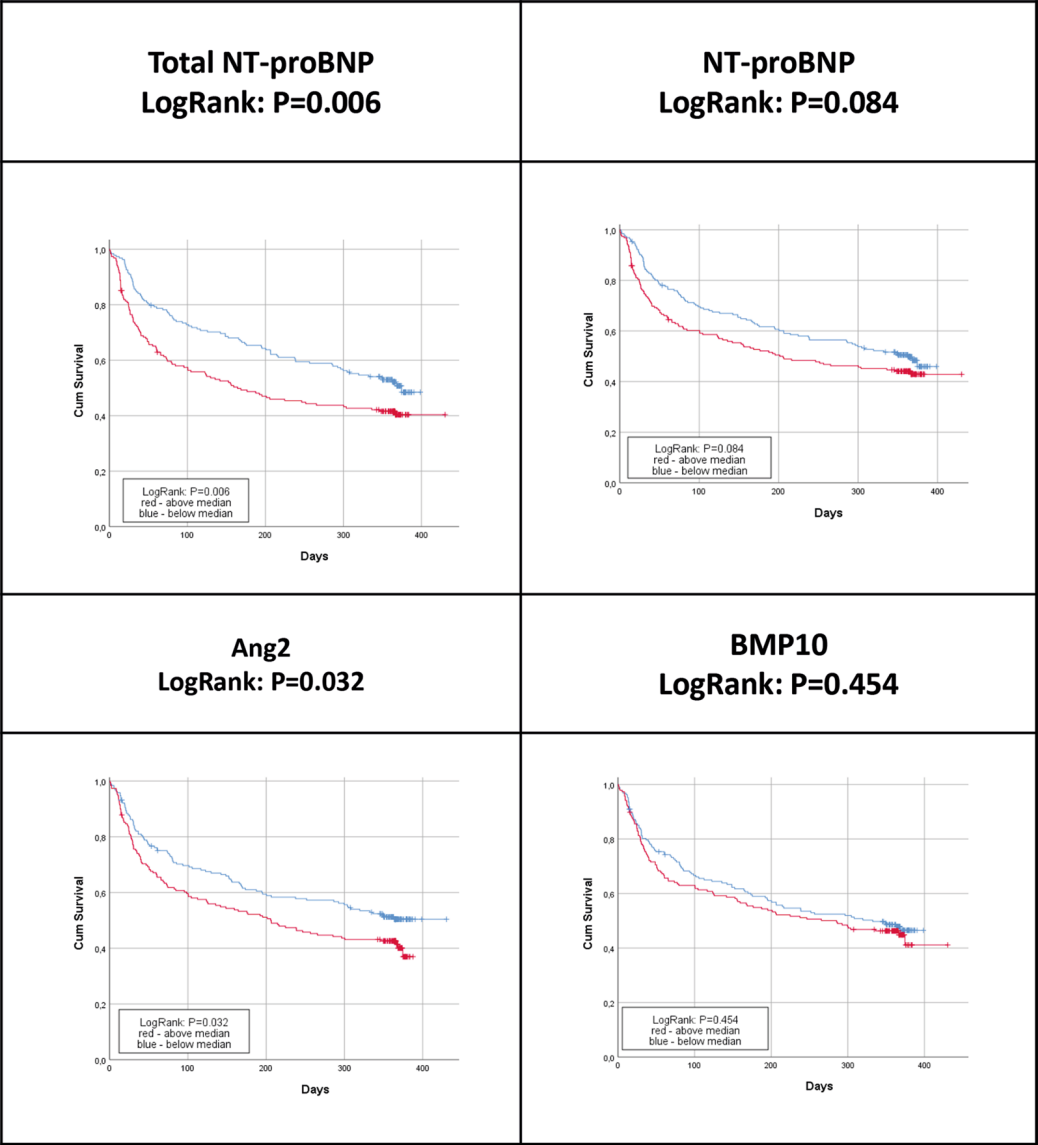
Kaplan–Meier curves of first episode of AF recurrence by median baseline biomarker concentration. Log rank test for: Total NT-proBNP, p = 0.006; NT-proBNP, p = 0.084; Ang2, p = 0.032 and BMP10, p = 0.454

**Fig. 2:**
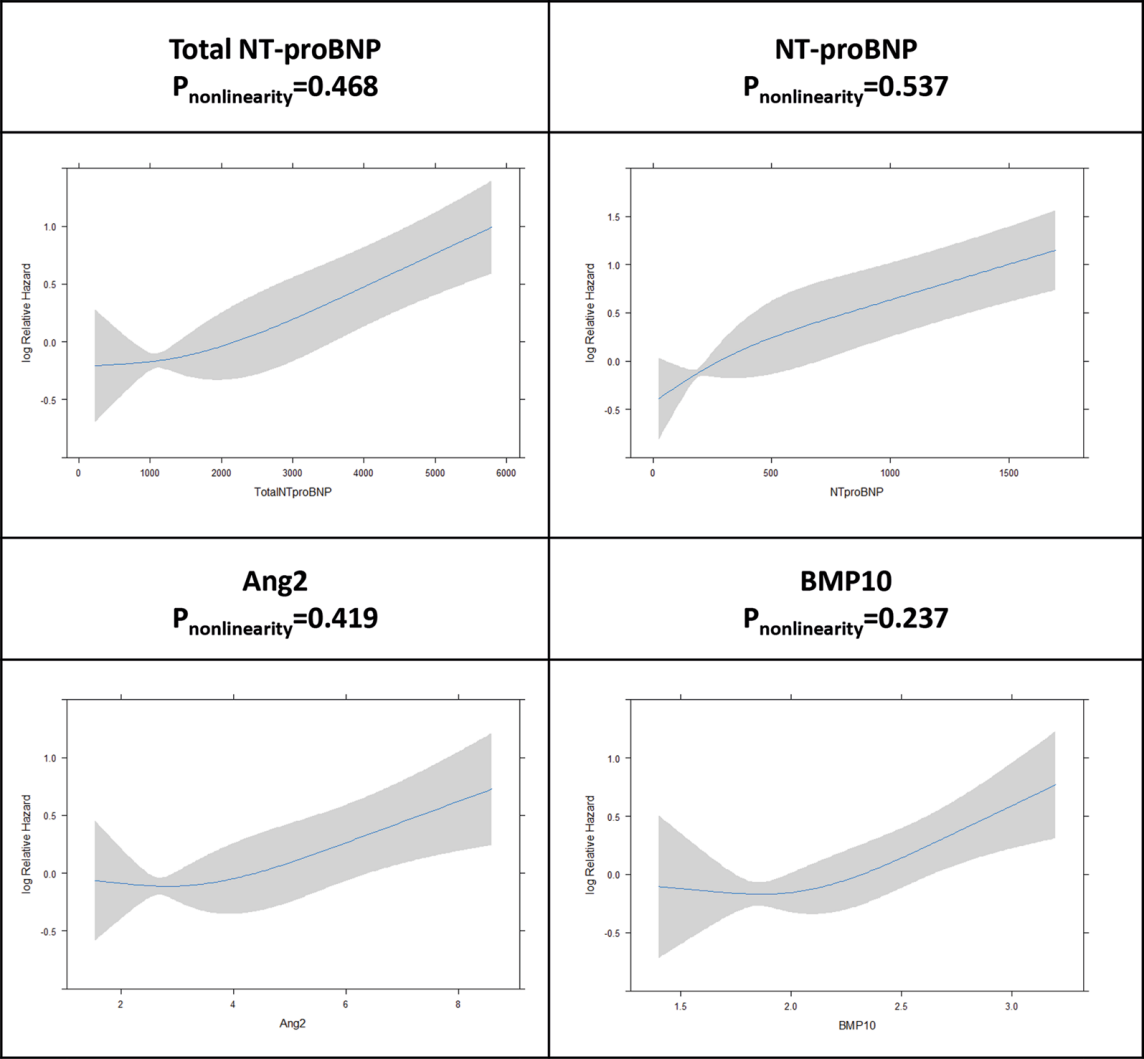
Restricted cubic spline depicting the continuous biomarker concentrations in relation to the risk of time to first AF recurrence. The continuous line indicates the central log relative hazard and the shaded area the 95% confidence intervals. P value for non-linearity: Total NT-proBNP, p = 0.362; NT-proBNP, p = 0.798; Ang2, p = 0.827 and BMP10, p = 0.364

Baseline concentrations of biomarkers in patients with or without AF recurrence were similar for all four biomarkers (Additional file 1: Table 3. Concentrations of circulating biomarkers at baseline in patients with or without AF recurrence). Kaplan Meier curves showing the incidence of AF recurrence in relation to the baseline biomarker concentration below or above the median indicate that curves started to diverge within the first week for total NT-proBNP, NT-proBNP and at one month for Ang2 and BMP10 (Fig. 1). Restricted cubic spline analysis showed linear associations for all four biomarkers with the recurrence of atrial fibrillation (Fig. 2), p > 0.05 for non-linearity. The predictive power for AF recurrence of baseline concentrations of total NT-proBNP, NT-proBNP, Ang2, and BMP10 was assessed using Cox proportional hazard models. After adjustment for clinical variables only total NT-proBNP (HR 1.19, 95%CI 1.04–1.36, p_fdr_ = 0.026) and NT-proBNP (HR 1.19, 95%CI 1.06–1.36, p_fdr_ = 0.016) significantly predicted AF recurrence (Table 4). The areas under the ROC curves based on the multivariate Cox models had a C-index of 0.822 for Total NT-proBNP and 0.829 for NT-proBNP, the difference was not statistically significant (P = 0.263) between the two models (Additional file 1: Fig. 1A ROC curves for first episode of recurrent atrial fibrillation. Curves are based on multivariable Cox survival model including standardized biomarkers – total-NT-proBNP and NT-proBNP – concentration). The risk of AF recurrence was analyzed in patients by quartiles of concentrations of the biomarkers at baseline. None of the quartiles were significantly different from the reference quartile after correction for multiple testing (Additional file 1: Table 4 Risk of AF recurrence for patients in the second, third and fourth quartiles of total-NT-proBNP, NT-proBNP, Ang2, BMP10 concentrations, compared to the lowest quartile.).

**Table 4:** Hazard ratios for atrial fibrillation recurrence according to candidate biomarkers

Biomarker	Univariate model		Multivariable model	
	HR (95%CI)	P_fdr_	HR (95%CI)	P_fdr_
Total NT-proBNP	1.15 (1.02–1.30)	**0.040**	1.19 (1.04–1.36)	**0.026**
NT-proBNP	1.21 (1.09–1.34)	**0.002**	1.19 (1.06–1.35)	**0.016**
Ang2	1.15 (1.01–1.30)	0.047	1.07 (0.95–1.20)	0.283
BMP10	1.01 (0.88–1.17)	0.898/	1.09 (0.96–1.25)	0.249

The prediction of AF recurrence was assessed by Cox proportional hazards regression models. Data shown as hazard ratio HR for an increment of 1 SD of baseline biomarker value. The multivariable model was adjusted for sex and > 2 episodes of AF in the six months before inclusion in the GISSI-AF trial. P-value was corrected for multiple testing by means of FDR-correction. HR, hazard ratio; NT-proBNP, N-terminal pro-B type natriuretic peptide; Ang2, angiopoietin 2; BMP10, bone morphogenic protein-10

### First hospitalization for CV reasons

Total NT-proBNP was associated with first hospitalization for CV reasons in multivariable Cox proportional hazards regression analysis (HR 1.57; 95%CI 1.29–1.90; p_fdr_ = 1.2 × 10^–5^); the association in this analysis was also significant for NT-proBNP after adjusting for confounders, (HR 1.57; 95%CI 1.34–1.84; p_fdr_ = 1.2 × 10^–6^) and Ang2 (HR 1.34; 95%CI 1.04–1.73; p = 0.029), but not for BMP10 (Table 5). The areas under the ROC curves based on the multivariate Cox models had a C-index of 0.725 for Total NT-proBNP and 0.742 for NT-proBNP, the difference was not statistically significant (P = 0.056) between the two models (Additional file 1: Fig. 1B ROC curves for first hospitalization for CV reasons. Curves are based on multivariable Cox survival model including standardized biomarkers – total-NT-proBNP and NT-proBNP – concentration). The risk of first hospitalization for CV reasons was significantly higher only for patients in the fourth quartile of NT-proBNP compared to those in the lowest quartile (Additional file 1: Table 5 Risk for first hospitalization for CV reasons for patients in the second, third and fourth quartiles of total-NT-proBNP, NT-proBNP, Ang2, BMP10 concentrations, compared to the lowest quartile.).

**Table 5:** Hazard ratios for first hospitalization for CV reasons

Biomarker	Univariate model		Multivariable model	
	HR [95%CI]	P_fdr_	HR [95%CI]	P_fdr_
Total NT-proBNP	1.54 (1.30–1.83)	**1.9 × 10**^**–6**^	1.57 (1.29–1.90)	**1.2 × 10**^**–5**^
NT-proBNP	1.49 (1.31–1.70)	**5.6 × 10**^**–9**^	1.57 (1.34–1.84)	**1.2 × 10**^**–6**^
Ang2	1.31 (1.08–1.59)	**0.007**	1.34 (1.04–1.73)	**0.029**
BMP10	1.38 (1.10–1.74)	**0.007**	1.25 (0.96–1.63)	0.097

The prediction of cardiovascular hospitalization was assessed by Cox proportional hazards regression. Data shown as hazard ratio HR for an increment of 1 SD of baseline biomarker concentration. The multivariable model was adjusted for systolic blood pressure, history of hypertension, peripheral artery disease and smoking. P-value was corrected for multiple testing by means of FDR-correction. HR, hazard ratio; NT-proBNP, N-terminal pro-B type natriuretic peptide; Ang2, angiopoietin 2; BMP10, bone morphogenic protein-10

## Discussion

This study in patients in sinus rhythm but at risk of AF shows for the first time that the relationship of ongoing AF and the risk of first AF recurrence and first hospitalization for CV reasons with the novel biomarker total NT-proBNP is as strong as that of NT-proBNP. From the panel of biomarkers studied, also BMP10, a marker of cardiomyocyte growth in the myocardium of the right atrium and ventricle, and Ang2, involved in inflammation and coagulation, were associated with AF.

While the association of a biomarker with AF may give useful mechanistic insights on the disease, a biomarker that can tell the doctor in advance what is the risk of a patient having new episodes of AF, is clinically important. That is why we assessed, for the first time, the association and the predictive power for AF of the plasma concentrations of total NT-proBNP in a cohort of patients with a history of AF, in sinus rhythm, at high risk of AF recurrence.

We hypothesized that the accuracy and prognostic value of NT-proBNP would improve using a biomarker to identify the glycosylated NT-proBNP. This task by itself is challenging, given the repeatedly reported superiority of NT-proBNP [7]. In fact, we did see that both total NT-proBNP and NT-proBNP gave significant and similar results for predicting AF. The median total NT-proBNP plasma concentration was 6.6, 7.2 and 6.8 times higher than NT-proBNP at baseline and at six- and 12-months follow-up (Table 1 and 2). The wide inter-individual variability in the ratio (e.g. 0.57 to 42.05) justifies the search for associations of total NT-proBNP and AF or clinical events. This indicated that in patients in sinus rhythm with a recent history of AF, NT-proBNP is extensively glycosylated and the extent of glycosylation does not change over time. In patients with acute dyspnea, and using deglycosylation enzymes to identify total NT-proBNP, Røsjø et al. [18] reported nearly double the levels of total NT-proBNP than NT-proBNP in HF and non-HF patients. At a median of 816 days, both natriuretic peptides were associated with all-cause mortality risk in HF patients; for the deglycosylated total NT-proBNP concentration the HR [95%CI] was 1.42 [1.24–1.63], p < 0.001, and for NT-proBNP 1.29 [1.13–1.46], p < 0.001. In the present study only 42 patients had clinically diagnosed HF or LVEF < 40% (11%); this small number does not permit analysis of AF stratified by HF, although the incidence of AF recurrence was no different in patients with HF (12.3%) and those without (9.5%, p = 0.38). Nonetheless, circulating biomarkers were significantly higher in patients with HF, independently of AF. The only exception was BMP10, apparently independent of HF (Additional file 1: Table 6 Concentrations of circulating biomarkers in patients with and without clinical HF or LVEF<40% ).

Ang2 showed a strong association with AF, confirming the pathogenic role of inflammatory activation in AF [19]; however, Ang2 had no predictive power for recurrent AF. In the GISSI-AF sub-study, similar results were reported for two other inflammatory markers, IL6 and hsCRP [6]. Like for Ang2 these two biomarkers were not independent predictors of AF recurrence.

In relation to AF, another marker closely involved in inflammations—GDF15—performed poorly in the present study. However, the ARISTOTLE large-scale trial reported GDF15 as a risk factor for major bleeding, mortality, and stroke in patients with permanent AF [20].

Very recently, in 359 patients after catheter ablation, BMP10 was an independent predictor of AF recurrence while NT-proBNP was not [16]. However, those patients were more severely ill than those in the present study (47% had HF and 12% a history of stroke; NT-proBNP approximately double), and absolute concentrations of BMP10 were only slightly higher.

Recently natriuretic peptides, particularly NT-proBNP, have been reported to be associated with inflammation [21]. This suggests a common pathophysiological mechanism for the biomarkers assessed in this study that were associated with AF.

### Strengths and limitations of the study

The main strengths of this study are: (a) it was a multicenter randomized clinical trial with concomitant serial echocardiography and circulating biomarkers analyzed centrally; and (b) trans-telephonic electrocardiographic monitoring enabled us to record and identify AF recurrence efficiently during the 12-month follow-up. Another strength is the simultaneous analysis of total NT-proBNP with other biomarkers involved in different pathophysiological mechanisms, some of them assessed for the first time for AF diagnosis or as predictors of AF recurrence (Ang2, BMP10). The potential added value of total NT-proBNP to the benchmark biomarker NT-proBNP was assessed from different dimensions of performance, as recently proposed for the evaluation of new biomarkers [20].

Our results cannot apply to all patients with AF since at baseline the GISSI-AF patients were in sinus rhythm and had a lower rate of co-morbidities than patients with AF in real life or in other cohorts with a higher frequency of persistent or permanent AF [1, 2, 18]. This was due to compliance with the strict eligibility criteria of the trial. The mean CHADS2 modified score was indeed very low, averaging 1.41 ± 0.84 in the whole population, compatible with the low morbidity rate of the patients selected [22]. The low frequency of deaths after 12 months (only one) and thromboembolic events (three) in the GISSI-AF sub-study could not be considered for the outcome analysis. The small number of patients with ongoing AF at a follow-up visit is a limitation for the association of the biomarkers with the diagnosis of AF.

## Conclusions

Total NT-proBNP performs similarly and is strongly correlated to NT-proBNP as a biomarker in AF. Total NT-proBNP and NT-proBNP were both significantly associated with an increased risk for first AF recurrence and first hospitalization for CV reasons. Ang2 and BMP10 were significantly associated with ongoing AF, pointing to a pathogenic role of cardiac extracellular matrix and cardiomyocyte growth in the myocardium of the right atrium and ventricle.

## Supplementary Information

**Additional file 1. Appendix 1** Detection of recurrent AF during follow-up. **Appendix 2** Assays of circulating biomarkers and detection of recurrent AF during follow-up. **Appendix 3** Participating centers and investigators. **Table 1** Baseline clinical, eletrocardiographic and echocardiograpic characteristics of all patients and according to ongoing AF and AF recurrence. **Table 2** Correlation coefficients for biomarker concentrations at baseline.**Table 3** Concentrations of circulating biomarkers at baseline in patients with or without AF recurrence. **Table 4** Risk of AF recurrence for patients in the second, third and fourth quartiles of total-NT-proBNP, NT-proBNP, Ang2, BMP10 concentrations, compared to the lowest quartile. **Table 5** Risk for first hospitalization for CV reasons for patients in the second, third and fourth quartiles of total-NTproBNP, NT-proBNP, Ang2, BMP10 concentrations, compared to the lowest quartile. **Table 6** Concentrations of circulating biomarkers in patients with and without clinical HF or LVEF<40%. **Fig. 1A** ROC curves for first episode of recurrent atrial fibrillation. Curves are based on multivariable Cox survival model including standardized biomarkers – total-NT-proBNP and NT-proBNP – concentration. **Fig. 1B** ROC curves for first hospitalization for CV reasons. Curves are based on multivariable Cox survival model including standardized biomarkers – total-NT-proBNP and NT-proBNP – concentration.

## Data Availability

The data are stored at the GISSI-AF Coordinating Centers (Florence and Milan). Data are available upon justified request to the GISSI-AF trial Steering Committee.

## Abbreviations

Ang2: Angiopoietin 2
BMP10: Bone morphogenic protein-10
CV: Cardiovascular
DKK3: Dickkopf-related protein 3
ELISA: Enzyme-linked immuno-sorbent assay
ESM1: Endothelial cell specific molecule 1
FABP3: Fatty acid binding protein 3
FGF23: Fibroblast growth factor 23
GDF15: Growth differentiation factor-15
HF: Heart failure
IGFBP7: Insulin-like growth factor-binding protein-7 (IGFBP7)
MyBPC3: Myosin binding protein C3
NT-proBNP: N-terminal pro-B type natriuretic peptide
