# Supplementary material for: Total NT-proBNP, a novel biomarker related to recurrent atrial fibrillation
Source: BMC Cardiovasc Disord. 2021 Nov 19;21:553. doi: 10.1186/s12872-021-02358-y (PMC8603582; doi:10.1186/s12872-021-02358-y)
Supplement: Supplementary file 1 — Additional file 1. Appendix 1 Detection of recurrent AF during follow-up. Appendix 2 Assays of circulating biomarkers and detection of recurrent AF during follow-up. Appendix 3 Participating centers and investigators. Table 1 Baseline clinical, eletrocardiographic and echocardiograpic characteristics of all patients and according to ongoing AF and AF recurrence. Table 2 Correlation coefficients for biomarker concentrations at baseline.Table 3 Concentrations of circulating biomarkers at baseline in patients with or without AF recurrence. Table 4 Risk of AF recurrence for patients in the second, third and fourth quartiles of total-NT-proBNP, NT-proBNP, Ang2, BMP10 concentrations, compared to the lowest quartile. Table 5 Risk for first hospitalization for CV reasons for patients in the second, third and fourth quartiles of total-NTproBNP, NT-proBNP, Ang2, BMP10 concentrations, compared to the lowest quartile. Table 6 Concentrations of circulating biomarkers in patients with and without clinical HF or LVEF<40%. Fig. 1A ROC curves for first episode of recurrent atrial fibrillation. Curves are based on multivariable Cox survival model including standardized biomarkers – total-NT-proBNP and NT-proBNP – concentration. Fig. 1B ROC curves for first hospitalization for CV reasons. Curves are based on multivariable Cox survival model including standardized biomarkers – total-NT-proBNP and NT-proBNP – concentration. [file 12872_2021_2358_MOESM1_ESM.doc]

**Additional file**

**Total NT-proBNP, a novel biomarker related to recurrent atrial fibrillation.**

Subtitle: Total-NT-proBNP in atrial fibrillation

Lidia Staszewsky^1*^, Jennifer M. T. A. Meessen^1*^, Deborah Novelli^1^, Ursula-Henrike Wienhues-Thelen^2^, Marcello Disertori^3^, Aldo P. Maggioni^4^, Serge Masson^5^, Gianni Tognoni^6^; Maria Grazia Franzosi^1^; Donata Lucci^4^, Roberto Latini^1^. *equally contributed to data analysis and interpretation.

^1^Department of Cardiovascular Medicine, Istituto di Ricerche Farmacologiche Mario Negri IRCCS, Milan, Italy; ^2^Roche Diagnostics GmbH, Penzberg, Germany, ^3^Healthcare Research and Innovation Program, IRCS-HTA, BK Foundation, Trento, Italy; ^4^ANMCO Research Center, Florence, Italy; ^5^Roche Diagnostics International Ltd, Rotkreuz, Switzerland;^6^Istituto di Anestesia e Rianimazione, Università degli Studi di Milano, Ospedale Maggiore, Istituto di Ricovero e Cura a Carattere Scientifico, Milan, Italy.

**Appendix 1** - Detection of recurrent AF during follow-up

**Appendix 2** - Assays of circulating biomarkers and detection of recurrent AF during follow-up.

**Appendix 3** *-* Participating centers and investigators

**Additional file Table 1**. Baseline clinical, eletrocardiographic and echocardiograpic characteristics of all patients and according to ongoing AF and AF recurrence.

**Additional file Table 2.** Correlation coefficients for biomarker concentrations at baseline.

**Additional file Table 3**. Concentrations of circulating biomarkers at baseline in patients with or without AF recurrence.

**Additional file Table 4.** Risk of AF recurrence for patients in the second, third and fourth quartiles of total-NT-proBNP, NT-proBNP, Ang2, BMP10 concentrations, compared to the lowest quartile.

**Additional file Table 5.** Risk for first hospitalization for CV reasons for patients in the second, third and fourth quartiles of total-NT-proBNP, NT-proBNP, Ang2, BMP10 concentrations, compared to the lowest quartile.

**Additional file Table 6**. Concentrations of circulating biomarkers in patients with and without clinical HF or LVEF<40%.

**Additional file Figure 1.** ROC curves for first episode of recurrent atrial fibrillation (A) and for first hospitalization for CV reasons (B). Curves are based on multivariable Cox survival model including standardized biomarkers concentration (total-NT-proBNP and NT-proBNP).

**Appendix 1 - Detection of recurrent AF during follow-up**

Recurrent AF was checked by electrocardiography at each study visit (day 1, weeks 2, 4, 8, 24 and 52). To increase the likelihood of detecting AF, patients were given a trans-telephonic monitoring device (Cardiobios 1, Telbios, Milan, Italy). The patient activated the tool transmitting a 30-s ECG to both the Study Coordinating Center and the attending physician in case of symptoms and every week during follow-up. Each episode of AF was assessed blindly by a central reader and verified by an ad-hoc committee (1).

**Appendix 2 - Assays of circulating biomarkers**

*Cardiac stress biomarkers*

- ***N-terminal pro-B type natriuretic peptide (NT-proBNP)*** is a pro-hormone synthetized by cardiomyocytes in response to: a) increased myocardial wall stress from volume and pressure load (2) and b) myocardial ischemia (3).
- ***Total N-terminal pro-B type natriuretic peptide (total NT-proBNP)***: this new pre-commercial Roche assay is also suitable for detection of glycosylated NT-proBNP. The term “total NT-proBNP” refers to the sum of glycosylated and unglycosylated NT-proBNP. Røsjø et al. (3) examined the influence of glycosylation on the diagnostic and prognostic accuracy of NT-proBNP in unselected patients with dyspnea, and found higher NT-proBNP concentrations after removing sugar moieties from NT-proBNP1-76 with deglycosylation enzymes in plasma from patients presenting dyspnea, particularly those with confirmed heart failure. The total NT-proBNP pre-commercial Roche assay overcomes the time-consuming, costly procedures of enzymatic deglycosylation used by Røsjø et al. (4)

*Markers of extracellular matrix*

- ***Angiopoietin 2 (Ang2)*** is a growth factor glycoprotein involved in angiogenesis, neovascularization, endothelial function and inflammatory processes (5)*.* Expression profiling studies have identified endothelial cells as the primary source of Ang 2. This angiopoietin is a transcriptionally powerfully regulated cytokine, stored in the Weibel-Palade bodies and acts in an autocrine manner to control endothelial cell dynamics. It can be secreted within a few minutes of stimulation, so is active in homeostatic reactions such as inflammation and coagulation (6).
- ***Dickkopf-related protein-3 (DKK3)***, expressed in the endothelium and muscles, is involved in the regulation of cardiac remodeling and differentiation of stem cells into vascular smooth muscle cells. It antagonizes cardiac hypertrophy by the Wnt signaling pathway (7).
- ***Bone morphogenic protein-10 (BMP10*)**: BMP-10 is a protein coding gene with a role in cardiomyocyte growth in the trabecular and compact myocardium of the right atrium and ventricle (8)*.* Circulating BMP10 is independently related to AF recurrence after ablation (9).
- ***Endothelial cell specific molecule-1 (ESM1) or Endocan*** , is a soluble

proteoglycan of 50 kDa, constituted of a mature polypeptide of 165 amino acids

and a single dermatan sulphate chain covalently linked to the serine residue at

position 137. ESM1, is expressed and secreted mainly by the vascular endothelium and its plasma concentration results increased in during cancer, sepsis, kidney diseases, renal transplant rejection, tumor progression and cardiovascular disease, suggesting that it could be a potential biomarker for endothelial activation or dysfunction (10).

- ***Fibroblast growth factor 23 (FGF23)*** is a hormone involved in phosphorus homeostasis, vitamin D metabolism and bone mineralization. It is a potential marker of renal function and stimulates fibroblast growth. Although it is still not clear how FGF23 is related to AF, its plasma concentration has been associated with endothelial dysfunction, vascular calcification and systemic inflammation (11,12).
- ***Growth differentiation factor-15 (GDF15)***, or macrophage inhibitory cytokine-1 (MIC-1), part of the transforming growth factor superfamily, is associated with other inflammatory markers. This marker is not cardio-specific, but has been reported to be related to impaired fibrin clot lysability in the left atrium (13).
- ***Insulin-like growth factor-binding protein-7 (IGFBP7)*** is expressed in many normal tissues and highly in the vasculature, particularly in Weibel-Palade bodies (14). It is involved in several physiological mechanisms such as cell proliferation, adhesion, senescence, collagen deposition, apoptosis and angiogenesis (15).

*Myocardial injury markers*

- ***Myosin binding protein (MyBPC3*)** is a thick nodular filament protein belonging to the intracellular Ig and fibronectin superfamily. It stabilizes the sarcomere structure and regulates actinomyosin cross-bridging; it is highly expressed in the cardiac muscle of the LV and is promptly released into the circulation after myocardial injury (16,17).
- ***Fatty acid-binding protein 3 (FABP3)*** - also termed heart-type fatty acid-binding protein - is a member of the intracellular lipid-binding protein family involved in fatty acid transport, cell growth, cellular signaling and gene transcription. FABP3 is highly expressed in cardiac and skeletal muscles and is released rapidly from the myocardium into the circulation after cardiomyocyte injury (18,19).

Laboratory personnel blinded to clinical information relating to the plasma samples did all the assays in Roche Diagnostics.

**Appendix 3**- **Participating centers and investigators**

*Switzerland*: Lugano (MG Rossi). *Italy*: Bagno a Ripoli (A Fazi), Bari Carbonara(O Pierfelice), Bergamo (A Gavazzi, F Taddei), Bovolone (G Rigatelli, S Boni), Bussolengo (R Trappolin), Casarano (A Muscella), Caserta (A Vetrano), Catania (M Gulizia,GM Francese), Catanzaro (F Perticone), Citta`di Castello (D Severini), Cremona (S Pirelli, A Spotti, M Mariani), Fidenza (P Pastori), Firenze (GM Santoro, C Minneci), NapoliFederico II (P Perrone Filardi), Palermo Cervello (L Buffa), Palermo Villa Sofia (F Ingrillı`),Pavia (L Tavazzi, C Belvito), Pesaro (A Pierantozzi), Pietra Ligure (A Nicolino), Reggio Calabria (G Pulitano`, A Ruggeri, G Cutrupi), Roma (M Volpe), Saluzzo (S Reynaud), SanBonifiacio (R Rossi, E Carbonieri, E Zampieri), San Daniele del Friuli (L Mos, G Marcuzzi),San Marco Argentano (O Cuccurullo), Sarzana (R Petacchi, D Bertoli), Terni(M Bernardinangeli, G Proietti, G Proietti), Trento Villa Bianca (G Cioffi, E Buczkowska),Trento Santa Chiara (P Zeni, C Giovannelli), Trieste ASS 1 (C Mazzone, D Radini), Trieste Università (A Aleksova).

**Additional file Table 1.** Baseline clinical, electrocardiographic and echocardiograpic characteristics of all patients and according to ongoing AF and AF recurrence.

|  | | All  (382) | AF during a study visit  (60) | No AF during a visit  (322) | P | Patients with  AF recurrence  (203) | Patients without AF recurrence  (179) | P |
| --- | --- | --- | --- | --- | --- | --- | --- | --- |
|  |  | n (%) | n (%) | n (%) |  | n (%) | n (%) |  |
| Female | | 142 (37.2) | 17 (28.3) | 125 (38.8) | 0.123 | 65 (32.0) | 77 (43.0) | 0.026 |
| Age, years, mean±SD | | 67.6±9.1 | 66.9+10.2 | 67.7+8.9 | 0.543 | 67.3±9.2 | 67.9±9.0 | 0.557 |
| BMI, kg/m2, mean±SD | | 27.9±4.3 | 28.3±3.8 | 27.8±4.4 | 0.408 | 27.9±3.9 | 27.8±4.7 | 0.838 |
| Systolic BP, mmHg, mean±SD | | 138.3±16.5 | 136.0±16.6 | 138.7±16.5 | 0.257 | 137.4±16.2 | 139.2±16.9 | 0.290 |
| Diastolic BP, mmHg, mean±SD | | 81.2±8.4 | 80.6±8.4 | 81.3±7.9 | 0.556 | 80.6±8.7 | 81.8±8.1 | 0.154 |
| eGFR, mLmin-^1^1.73m^-2^, mean±SD | | 76.3±25.0 | 77.7±24.7 | 76.1±25.1 | 0.650 | 78.1±25.6 | 74.3±24.3 | 0.142 |
|  | | | | | | | | |
| >2 episodes in AF, prior 6 months | | 154 (40.8) | 21 (35.6) | 133 (41.8) | 0.371 | 93 (46.3) | 61 (34.7) | 0.022 |
| Cardioversion, prior 2 weeks | | 336 (88.0) | 56 (93.3) | 280 (87.0) | 0.163 | 174 (85.7) | 162 (90.5) | 0.151 |
| Heart failure. LVEF<40% or both | | 42 (11.0) | 14 (23.3) | 28 (8.7) | 0.001 | 25 (12.3) | 17 (9.5) | 0.380 |
| History of hypertension | | 324 (84.8) | 41 (68.3) | 283 (87.9) | <0.0001 | 167 (82.3) | 157 (87.7) | 0.139 |
| Diabetes mellitus | | 50 (13.1) | 10 (16.7) | 40 (12.4) | 0.371 | 28 (13.8) | 22 (12.3) | 0.664 |
| History of stroke | | 15 (3.9) | 1 (1.7) | 14 (4.3) | 0.326 | 7 (3.4) | 8 (4.5) | 0.608 |
| Peripheral artery disease | | 22 (5.8) | 6 (10.0) | 16 (5.0%) | 0.125 | 15 (7.4) | 7 (3.9) | 0.145 |
| Documented CAD | | 41 (10.7) | 7 (11.7) | 34 (10.6) | 0.799 | 25 (12.3) | 16 (8.9) | 0.287 |
| AF episode with LA dilatation | | 52 (13.6) | 16 (26.7) | 36 (11.2) | 0.001 | 33 (16.) | 19 (10.6) | 0.109 |
|  | | | | | | | | |
| Peripheral embolism | | 4 (1.0) | 0 | 4 (1.2) | 0.385 | 3 (1.5) | 1 (0.6) | 0.378 |
| Renal dysfunction | | 10 (2.6) | 2 (3.3) | 8 (2.5) | 0.705 | 5 (2.5) | 5 (2.8) | 0.840 |
| COPD | | 33 (8.6) | 6 (10.0) | 27 (8.4%) | 0.683 | 21 (1.3) | 12 (6.) | 0.206 |
| Neoplasia | | 12 (3.15) | 0 | 12 (3.) | 0.129 | 4 (2.0) | 8 (4.5) | 0.162 |
| Current smoking | | 36 (9.4) | 7 (11.7) | 29 (9.0) | 0.371 | 20 (9.9) | 16 (8.9) | 0.212 |
| Alcohol abuse | | 4 (1.0) | 1 (1.7) | 3 (0.9) | 0.608 | 1 (0.5) | 3 (1.7) | 0.257 |
|  | | | | | | | | |
| Heart rate, mean±SD | 381 | 62.2±9.7 | 62.3±9.3 | 62.2±9.8 | 0.983 | 61.9±9.8 | 62.6±9.6 | 0.496 |
| QRS > 120 ms | 381 | 43 (11.3) | 8 (13.3) | 35 (10.9) | 0.585 | 23 (11.4) | 20 (11.2) | 0.948 |
| LVH | 382 | 34 (8.9) | 7 (11.7) | 27 (8.4) | 0.412 | 19 (9.) | 15 (8.4) | 0.737 |
| Pathological Q waves | 382 | 11 (2.9) | 3 (5.0) | 8 (2.5) | 0.285 | 6 (3.0) | 5 (2.8) | 0.925 |
|  | | | | | | | | |
| LAVI_max_, mean±SD | 283 | 42.6±14.7 | 49.8±16.3 | 41.4±14.1 | 0.001 | 43.6±16.0 | 41.5±13.2 | 0.246 |
| LAVI_min_, mean±SD | 277 | 23.3±12.3 | 30.9 14.3 | 22.0±11.4 | <0.0001 | 24.5±13.4 | 22.0±10.8 | 0.092 |
| LAEF, mean±SD | 275 | 47.5±13.3 | 39.4± 11.6 | 49.0±13.1 | <0.0001 | 46.6±13.9 | 48.6±12.5 | 0.224 |
| E/e, mean±SD’ | 108 | 12.6±8.6 | 13.±8.0 | 12.4±8.8 | 0.682 | 13.7± 1.3 | 11.5±4.8 | 0.191 |
| LVEF, mean±SD | 289 | 60.5±12.3 | 53.8 13.7 | 61.7±11.7 | <0.0001 | <0.0001 | 61.6±11.3 | 0.171 |
|  | | | | | | | | |
| Amiodarone | | 148 (38.7) | 29 (48.9) | 119 (37.0) | 0.097 | 74 (36.) | 74 (41.3) | 0.328 |
| Sotalol | | 28 (7.3) | 7 (11.7) | 21 (6.5) | 0.160 | 18 (8.9) | 10 (5.6) | 0.220 |
| ACE inhibitors | | 206 (53.9) | 37 (61.8) | 169 (52.5) | 0.190 | 115 (56.7) | 91 (50.8) | 0.255 |
| Beta-blockers | | 114 (29.8) | 18 (30.0) | 96 (29.8) | 0.977 | 58 (28.) | 56 (31.3) | 0.563 |
| Digitalis | | 16 (4.2) | 4 (6.7) | 12 (3.7) | 0.297 | 10 (4.9) | 6 (3.4) | 0.443 |
| Diuretics | | 16 (4.2) | 26 (43.3) | 123 (38.2) | 0.454 | 10 (4.9%) | 6 (3.4) | 0.220 |
| Aldosterone blockers | | 20 (5.2) | 3 (5.0) | 17 (5.3) | 0.929 | 8 (3.9) | 12 (6.7) | 0.226 |
| Statins | | 98 (25.7) | 14 (23.3) | 84 (26.1) | 0.654 | 53 (26.) | 45 (14.1) | 0.829 |
| Oral anticoagulants | | 233 (61.0) | 52 (86.7) | 181 (56.2) | <0.0001 | 126 (62.1) | 107 (59.8) | 0.647 |
| Aspirin | | 101 (26.4) | 5 (8.3) | 96 (29.8) | 0.001 | 55 (27.1) | 46 (25.7) | 0.758 |
| Randomized to Valsartan | | 186 (48.7) | 32 (53.3) | 154 (47.8) | 0.433 | 106 (52.2) | 80 (44.7) | 0.142 |
| Table contents: modified from Latini et al. JIM 2010 previous author’s authorization.  COPD - chronic obstructive pulmonary disease; LVH - left ventricular hypertrophy; LAVI_max_ - left atrial maximal volume index; LAVI_min_ - left atrial minimum volume index; LAEF - left atrial emptying fraction; E/e’ - ratio of early transmitral Doppler flow velocity to peak early diastolic tissue velocity of the septal mitral annulus; LVEF - left ventricular ejection fraction. | | | | | | | | |

**Additional file Table 2. Correlation coefficients for biomarker concentrations at baseline.**

|  | TOTAL NTproBNP (pg/mL) | NTproBNP (pg/mL) | Ang2 (ng/mL) | BMP10  (ng/mL) | DKK3  (ng/mL) | ESM1  (ng/mL) | FABP3  (ng/mL) | FGF23 (ng/mL) | GDF15  (pg/mL) | IBP7  (ng/mL) | MYBPC3  (pg/mL) |
| --- | --- | --- | --- | --- | --- | --- | --- | --- | --- | --- | --- |
| TOTAL NT-proBNP (pg/mL) | 1.00 |  |  |  |  |  |  |  |  |  |  |
| NT-proBNP (pg/mL) | 0.90 | 1.00 |  |  |  |  |  |  |  |  |  |
| Ang2 (ng/mL) | 0.49 | 0.54 | 1.00 |  |  |  |  |  |  |  |  |
| BMP10  (ng/mL) | 0.46 | 0.43 | 0.29 | 1.00 |  |  |  |  |  |  |  |
| DKK3  (ng/mL) | 0.48 | 0.43 | 0.29 | 0.53 | 1.00 |  |  |  |  |  |  |
| ESM1  (ng/mL) | 0.41 | 0.38 | 0.19 | 0.44 | 0.53 | 1.00 |  |  |  |  |  |
| FABP3  (ng/mL) | 0.34 | 0.32 | 0.11 | 0.29 | 0.32 | 0.19 | 1.00 |  |  |  |  |
| FGF23 (pg/mL) | 0.32 | 0.36 | 0.29 | 0.26 | 0.16 | 0.16 | 0.27 | 1.00 |  |  |  |
| GDF15  (pg/mL) | 0.54 | 0.55 | 0.33 | 0.37 | 0.40 | 0.33 | 0.49 | 0.33 | 1.00 |  |  |
| IGFBP7  (ng/mL) | 0.56 | 0.49 | 0.42 | 0.47 | 0.49 | 0.35 | 0.46 | 0.42 | 0.59 | 1.00 |  |
| MYBPC3  (ng/mL) | 0.54 | 0.58 | 0.35 | 0.31 | 0.37 | 0.27 | 0.34 | 0.31 | 0.49 | 0.49 | 1.00 |
| Spearman correlation coefficients (r). NT-proBNP - N-terminal pro-B type natriuretic peptide; Ang2 - angiopoietin 2; BMP10 - bone morphogenic protein-10; DKK3 - Dickkopf-related protein-3; ESM1 - endothelial cell-specific molecule 1; FABP3 - fatty acid-binding protein 3; FGF23 - fibroblast growth factor 23; GDF15 - growth differentiation factor-15; IGFBP7 - insulin like growth factor-binding protein-7; MyBPC3 - myosin binding protein C3. | | | | | | | | | | | |

**Additional file Table 3.** Concentrations of circulating biomarkers at baseline in patients with and without AF recurrence during follow-up.

| . | **Total**  (382) | | **No AF recurrence**  (179) | | **AF recurrence**  (203) | | **P *** |
| --- | --- | --- | --- | --- | --- | --- | --- |
|  | Median | IQR | Median | IQR | Median | IQR |  |
| Total NT-proBNP, pg/mL | 1230 | 667-2011 | 1110 | 651-1882 | 1355 | 680-2142 | 0.139 |
| NT-proBNP, pg/mL | 191 | 94.8-367 | 183 | 89-225 | 207 | 99-458 | 0.159 |
| Ang2, ng/mL | 2.91 | 2.21-3.99 | 2.66 | 2.19-3.76 | 3.08 | 2.22-4.18 | 0.152 |
| BMP10, pg/mL | 2.02 | 1.75-2.33 | 2.01 | 1.77-2.37 | 2.04 | 1.71-2.29 | 0.636 |
| * P value for Mann-Whitney test. NT-proBNP - N-terminal pro-B type natriuretic peptide; Ang2 - angiopoietin 2; BMP10 - bone morphogenic protein-10. | | | | | | | |

**Additional file Table 4.** Risk for AF recurrence in patients in the second, third and fourth quartiles of total-NT-proBNP, NT-proBNP, Ang2, BMP10 concentrations, compared to the lowest quartile.

| Biomarker | quartile | Univariate model | | Multivariable model | |
| --- | --- | --- | --- | --- | --- |
|  |  | HR [95%CI] | P | HR [95%CI] | P |
| Total NT-proBNP | 1 - ref |  |  |  |  |
|  | 2 | 0.86 [0.57-1.30] | 0.474 | 1.07 [0.71-1.61] | 0.760 |
|  | 3 | 1.37 [0.93-2.02] | 0.170 | 1.61 [1.09-2.39] | 0.054 |
|  | 4 | 1.38 [0.94-2.03] | 0.170 | 1.40 [0.95-2.07] | 0.131 |
| NT-proBNP | 1 – ref |  |  |  |  |
|  | 2 | 1.13 [0.76-1.69] | 0.539 | 1.10 [0.74-1.64] | 0.643 |
|  | 3 | 1.20 [0.80-1.79] | 0.539 | 1.38 [0.92-2.06] | 0.185 |
|  | 4 | 11.52 [1.03-2.23] | 0.099 | 1.44 [0.98-2.12] | 0.185 |
| Ang 2 | 1 – ref |  |  |  |  |
|  | 2 | 0.79 [0.53-1.20] | 0.405 | 0.91 [0.60-1.38] | 0.660 |
|  | 3 | 1.27 [0.87-1.85] | 0.405 | 1.25 [0.85-1.82] | 0.632 |
|  | 4 | 1.15 [0.78-1.70] | 0.470 | 1.17 [0.80-1.73] | 0.632 |
| BMP10 | 1 - ref |  |  |  |  |
|  | 2 | 0.70 [0.47-1.05] | 0.258 | 1.01 [0.67-1.52] | 0.973 |
|  | 3 | 1.10 [0.76-1.58] | 0.630 | 1.42 [0.97-2.06] | 0.213 |
|  | 4 | 0.80 [0.54-1.19] | 0.407 | 1.24 [0.83-1.87] | 0.449 |
| AF recurrence was predicted by Cox proportional hazards regression models including quartiles of biomarker concentration. The multivariable model was adjusted for sex and two or more episodes of AF in the six months before inclusion in the GISSI-AF trial. NT-proBNP, N-terminal pro-B type natriuretic peptide; Ang 2, angiopoietin 2; BMP10, bone morphogenic protein-10. P-value corrected for multiple testing by FDR-correction. | | | | | |

**Additional file Table 5.** Risk for first hospitalization for CV reasons, in patients in the second, third and fourth quartiles of total-NT-proBNP, NT-proBNP, Ang2, BMP10 concentrations, compared to the lowest quartile.

| Biomarker | quartile | Univariate model | | Multivariable model | |
| --- | --- | --- | --- | --- | --- |
|  |  | HR [95%CI] | P | HR [95%CI] | P |
| Total NT-proBNP | 1 - ref |  |  |  |  |
|  | 2 | 0.58 [0.23-1.48] | 0.383 | 0.55 [0.20-1.50] | 0.365 |
|  | 3 | 1.12 [0.51-2.46] | 0.773 | 1.12 [0.49-2.56] | 0.782 |
|  | 4 | 2.05 [1.02-4.13] | 0.129 | 1.89 [0.88-4.06] | 0.312 |
| NT-proBNP | 1 – ref |  |  |  |  |
|  | 2 | 1.36 [0.57-3.22] | 0.598 | 1.30 [0.53-3.21] | 0.828 |
|  | 3 | 1.27 [0.53-3.06] | 0.598 | 1.11 [0.44-2.82] | 0.828 |
|  | 4 | **3.08 [1.44-6.60]** | **0.012** | **2.96 [1.34-6.52]** | **0.021** |
| Ang 2 | 1 – ref |  |  |  |  |
|  | 2 | 0.92 [0.42-2.03] | 0.843 | 1.30 [0.57-2.99] | 0.807 |
|  | 3 | 0.86 [0.38-1.91] | 0.843 | 1.03 [0.44-2.43] | 0.947 |
|  | 4 | 1.64 [0.82-3.30] | 0.495 | 1.68 [0.77-3.66] | 0.579 |
| BMP10 | 1 - ref |  |  |  |  |
|  | 2 | 0.75 [0.33-1.72] | 0.581 | 0.67 [0.28-1.62] | 0.974 |
|  | 3 | 1.23 [0.59-2.55] | 0.581 | 1.16 [0.55-2.49] | 0.974 |
|  | 4 | 1.26 [0.61-2.62] | 0.581 | 0.99 [0.45-2.18] | 0.974 |
| The first hospitalization was predicted by Cox proportional hazards regression models including quartiles of biomarker concentration. The multivariable model was adjusted for systolic blood pressure, history of hypertension, peripheral artery disease and smoking. NT-proBNP, N-terminal pro-B type natriuretic peptide; Ang 2, angiopoietin 2; BMP10, bone morphogenic protein-10. P-value corrected for multiple testing by FDR-correction. | | | | | |

**Additional file Table 6**. Concentrations of circulating biomarkers in patients with and without clinical HF or LVEF<40%.

|  | | **Total**  **382** | | **Heart failure or LVEF<40%**  **42** | | **No heart failure**  **340** | | **P *** |
| --- | --- | --- | --- | --- | --- | --- | --- | --- |
|  |  | Median | IQR | Median | IQR | Median | IQR |  |
| Total NT-proBNP  (pg/mL) | baseline | 1230 | 667-2011 | 1962 | 1070-4212 | 1129 | 647-1913 | <0.001 |
|  | 6 months | 985 | 507-1959 | 1546 | 563-3289 | 939 | 501-1855 | 0.021 |
|  | 12 months | 1008 | 564-2112 | 1456 | 704-3352 | 976 | 541-2051 | 0.100 |
| NT-proBNP  (pg/mL) | baseline | 191 | 95-367 | 436 | 214-851 | 178 | 89-316 | <0.001 |
|  | 6 months | 136 | 66-324 | 222 | 95-730 | 133 | 59-302 | 0.006 |
|  | 12 months | 149 | 63-350 | 296 | 115-809 | 140 | 61-327 | 0.007 |
| Ang2 (ng/mL) | baseline | 2.91 | 2.21-3.99 | 3.95 | 2.91-5.78 | 2.77 | 2.16-3.71 | <0.001 |
|  | 6 months | 2.49 | 2.01-3.19 | 3.03 | 2.36-4.55 | 2.47 | 1.98-3.07 | 0.005 |
|  | 12 months | 2.56 | 2.02-3.28 | 2.85 | 2.30-4.91 | 2.53 | 1.97-3.22 | 0.005 |
| BMP10 (ng/mL) | baseline | 2.02 | 1.75-2.33 | 2.06 | 1.82-2.46 | 2.02 | 1.73-2.33 | 0.377 |
|  | 6 months | 2.01 | 1.75-2.28 | 2.00 | 1.81-2.33 | 2.01 | 1.72-2.28 | 0.631 |
|  | 12 months | 2.00 | 1.74-2.34 | 1.95 | 1.64-2.26 | 2.02 | 1.77-2.34 | 0.380 |
| * Kruskal-Wallis P value. NT-proBNP - N-terminal pro-B type natriuretic peptide; Ang2 - angiopoietin 2; BMP10 - bone morphogenic protein-10. P-value corrected for multiple testing by FDR-correction. | | | | | | | | |

**Additional file Figure 1**. **Additional file Figure 1**. ROC curves for first episode of recurrent atrial fibrillation (A) and for first hospitalization for CV reasons (B). Curves are based on multivariable Cox survival model including standardized biomarkers concentration (total-NT-proBNP and NT-proBNP). Cox analysis for first episode of recurrent atrial fibrillation was adjusted for sex and ≥2 episodes of AF in previous six months for AF recurrence and that for first hospitalization for CV reasons was adjusted for systolic blood pressure, history of hypertension, peripheral artery disease and smoking.


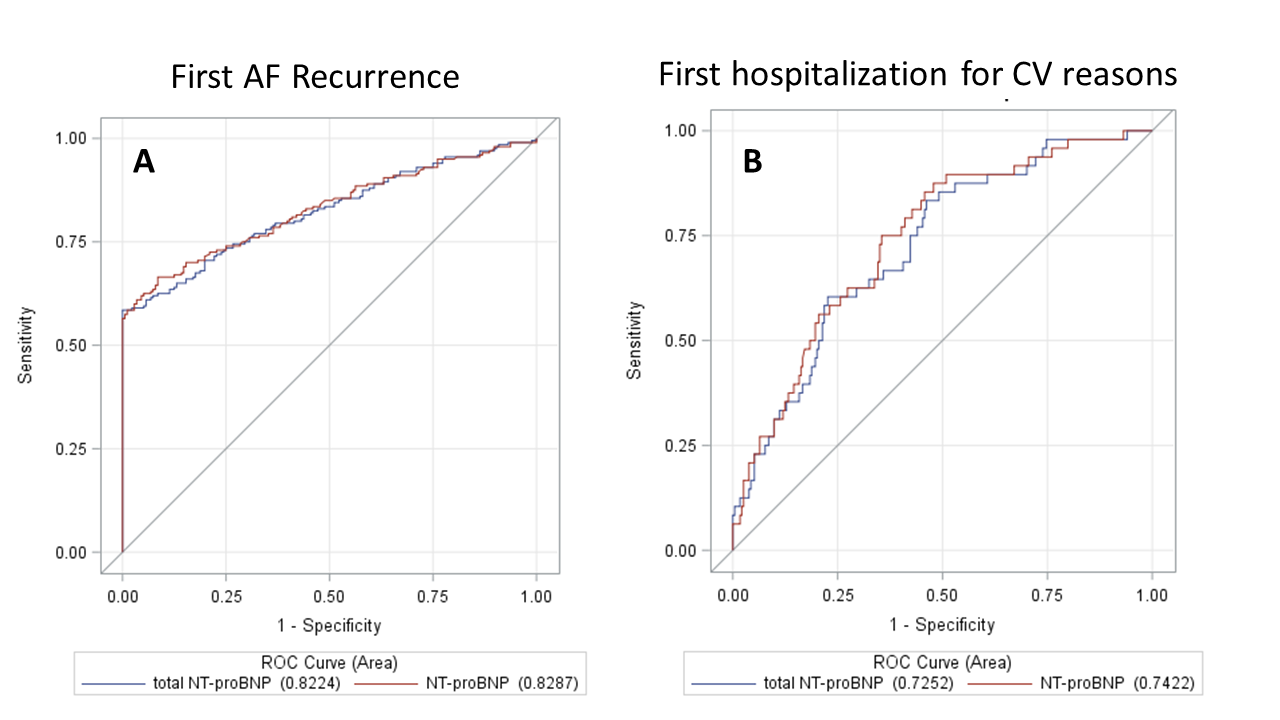


**Additional file REFERENCES**

1. Disertori M, Latini R, Maggioni AP, Delise P, Di Pasquale G, Franzosi MG, et al. Rationale and design of the GISSI-Atrial Fibrillation Trial: a randomized, prospective, multicentre study on the use of valsartan, an angiotensin II AT1-receptor blocker, in the prevention of atrial fibrillation recurrence. J Cardiovasc Med (Hagerstown). 2006 Jan;7(1):29–38.

2. Levin ER, Gardner DG, Samson WK. Natriuretic peptides. N Engl J Med. 1998 Jul 30;339(5):321–8.

3. Goetze JP, Christoffersen C, Perko M, Arendrup H, Rehfeld JF, Kastrup J, et al. Increased cardiac BNP expression associated with myocardial ischemia. FASEB J. 2003 Jun;17(9):1105–7.

4. Røsjø H, Dahl MB, Jørgensen M, Røysland R, Brynildsen J, Cataliotti A, et al. Influence of glycosylation on diagnostic and prognostic accuracy of N-terminal pro-B-type natriuretic peptide in acute dyspnea: data from the Akershus Cardiac Examination 2 Study. Clin Chem. 2015 Aug;61(8):1087–97.

5. Freestone B, Chong AY, Lim HS, Blann A, Lip GYH. Angiogenic factors in atrial fibrillation: a possible role in thrombogenesis? Ann Med. 2005;37(5):365–72.

6. Fiedler U, Scharpfenecker M, Koidl S, Hegen A, Grunow V, Schmidt JM, et al. The Tie-2 ligand angiopoietin-2 is stored in and rapidly released upon stimulation from endothelial cell Weibel-Palade bodies. Blood. 2004 Jun 1;103(11):4150–6.

7. Mathew JS, Sachs MC, Katz R, Patton KK, Heckbert SR, Hoofnagle AN, et al. Fibroblast growth factor-23 and incident atrial fibrillation: the Multi-Ethnic Study of Atherosclerosis (MESA) and the Cardiovascular Health Study (CHS). Circulation. 2014 Jul 22;130(4):298–307.

8. Chua W, Easter CL, Guasch E, Sitch A, Casadei B, Crijns HJGM, et al. Development and external validation of predictive models for prevalent and recurrent atrial fibrillation: a protocol for the analysis of the CATCH ME combined dataset. BMC Cardiovasc Disord. 2019 21;19(1):120.

9. van Breevoort D, van Agtmaal EL, Dragt BS, Gebbinck JK, Dienava-Verdoold I, Kragt A, et al. Proteomic screen identifies IGFBP7 as a novel component of endothelial cell-specific Weibel-Palade bodies. Journal of Proteome Research. 2012 May 4;11(5):2925–36.

10. Gandhi PU, Chow SL, Rector TS, Krum H, Gaggin HK, McMurray JJ, et al. Prognostic Value of Insulin-Like Growth Factor-Binding Protein 7 in Patients with Heart Failure and Preserved Ejection Fraction. J Card Fail. 2017 Jan;23(1):20–8.

11. Yu B, Kiechl S, Qi D, Wang X, Song Y, Weger S, et al. A Cytokine-Like Protein Dickkopf-Related Protein 3 Is Atheroprotective. Circulation. 2017 Sep 12;136(11):1022–36.

12. Kahr PC, Piccini I, Fabritz L, Greber B, Schöler H, Scheld HH, et al. Systematic analysis of gene expression differences between left and right atria in different mouse strains and in human atrial tissue. PLoS ONE. 2011;6(10):e26389.

13. Reyat JS, Chua W, Cardoso VR, Witten A, Kastner PM, Kabir SN, et al. Reduced left atrial cardiomyocyte PITX2 and elevated circulating BMP10 predict atrial fibrillation after ablation. JCI Insight. 2020 Aug 20;5(16).

14. Sarrazin S, Adam E, Lyon M, Depontieu F, Motte V, Landolfi C, Lortat-Jacob H, Bechard D, Lassalle P, Delehedde M. Endocan or endothelial cell specific molecule-1 (ESM-1): a potential novel endothelial cell marker and a new target for cancer therapy. Biochim Biophys Acta. 2006 Jan;1765(1):25-37. doi:10.1016/j.bbcan.2005.08.004. Epub 2005 Aug 26. PMID: 16168566.

15. Wallentin L, Hijazi Z, Andersson U, Alexander JH, De Caterina R, Hanna M, et al. Growth differentiation factor 15, a marker of oxidative stress and inflammation, for risk assessment in patients with atrial fibrillation: insights from the Apixaban for Reduction in Stroke and Other Thromboembolic Events in Atrial Fibrillation (ARISTOTLE) trial. Circulation. 2014 Nov 18;130(21):1847–58.

16. Kuster DWD, Cardenas-Ospina A, Miller L, Liebetrau C, Troidl C, Nef HM, et al. Release kinetics of circulating cardiac myosin binding protein-C following cardiac injury. Am J Physiol Heart Circ Physiol. 2014 Feb 15;306(4):H547-556.

17. Lahm H, Dreßen M, Beck N, Doppler S, Deutsch M-A, Matsushima S, et al. Myosin binding protein H-like (MYBPHL): a promising biomarker to predict atrial damage. Sci Rep. 2019 10;9(1):9986.

18. Smathers RL, Petersen DR. The human fatty acid-binding protein family: evolutionary divergences and functions. Hum Genomics. 2011 Mar;5(3):170–91.

19. Shirakabe A, Hata N, Kobayashi N, Okazaki H, Matsushita M, Shibata Y, et al. Worsening renal failure in patients with acute heart failure: the importance of cardiac biomarkers. ESC Heart Fail. 2019 Apr;6(2):416–27.
